# ML-based electromyography signal analysis for assessing rehabilitation exercise execution quality

**DOI:** 10.1038/s41598-026-59615-3

**Published:** 2026-07-02

**Authors:** Finn Siegel, Andreas Hein, Matthias Maszuhn, Anna Schumacher

**Affiliations:** 1 Department of Health Services Research, Assistance Systems and Medical Device Technology, Carl von Ossietzky Universität Oldenburg, Ammerländer Heerstr, 114- 118, 26129 Oldenburg, Germany; 2 OFFIS e.V.- Institute for Information Technology, Escherweg 2, Oldenburg, Germany

**Keywords:** Electromyography, Rehabilitation, Machine learning, Classification, Novelty detection, Engineering, Health care, Mathematics and computing, Neuroscience

## Abstract

Following lower limb trauma, performing orthopaedic rehabilitation exercises is a crucial factor in successful recovery. However, many patients find it difficult to execute these movements with optimal biomechanical form. To address this problem, an electromyography-based sensor system combined with machine learning is proposed. This system records and analyses exercises performed with the aim of providing real-time feedback on execution quality. To evaluate the potential of this concept, high-density electromyographic data (32 electrodes per sensor pad) were recorded from the vastus lateralis and vastus medialis muscles while performing rehabilitation exercises. Four different exercises (squat, hip abduction, leg raises and rocking) were performed, each in one optimal and three non-optimal variations, by *n* = 19 participants, resulting in 3,040 exercise executions and 194,560 electromyographic recordings. Analysis of this dataset demonstrated that a Support Vector Machine algorithm can be used to classify execution quality (four classes per exercise) with an average accuracy of 83.3% (± 8.8%). In addition, it was shown that, One Class Support Vector Machine trained with solely optimal executions, an unknown exercise could be identified as either optimal or non-optimal execution with an accuracy of 76.6% (± 5.9%). These results highlight the potential of this approach to evaluate exercise execution quality during rehabilitation. In the long term, this approach could provide personalised rehabilitation feedback and improve patient outcome.

## Introduction

Orthopaedic Rehabilitation following trauma of the lower limb plays a critical role in ensuring effective recovery and restoring physical function^1,2^. The effectiveness of rehabilitative interventions depends strongly on adherence to the prescribed exercises^3,4^. Failure to comply with the prescribed rehabilitation measures can have serious consequences, including delayed or impaired healing, physical deterioration, increased risk of thromboembolic events, pneumonia or decubitus^5–7^.

Initially, the rehabilitation process is overseen by experts, who supervise the progress and intervene when necessary. As recovery progresses, the level of expert support declines, and the responsibility to maintain the rehabilitation plan is transferred to the patient. During this phase the risk of suboptimal continuation rises, potentially leading to a prolonged recovery^6,8^. Research has highlighted widespread issues of poor adherence during this phase, with studies showing that fewer than half of patients adhere to prescribed exercises^5,9^. One potential solution for optimising the adherence to a rehabilitation plan is digital support for patients.

Sensor technology can be used to provide feedback on quantity and quality of exercises performed, which can increase adherence and improve rehabilitation outcomes^10,11^.

**Fig. 1:**
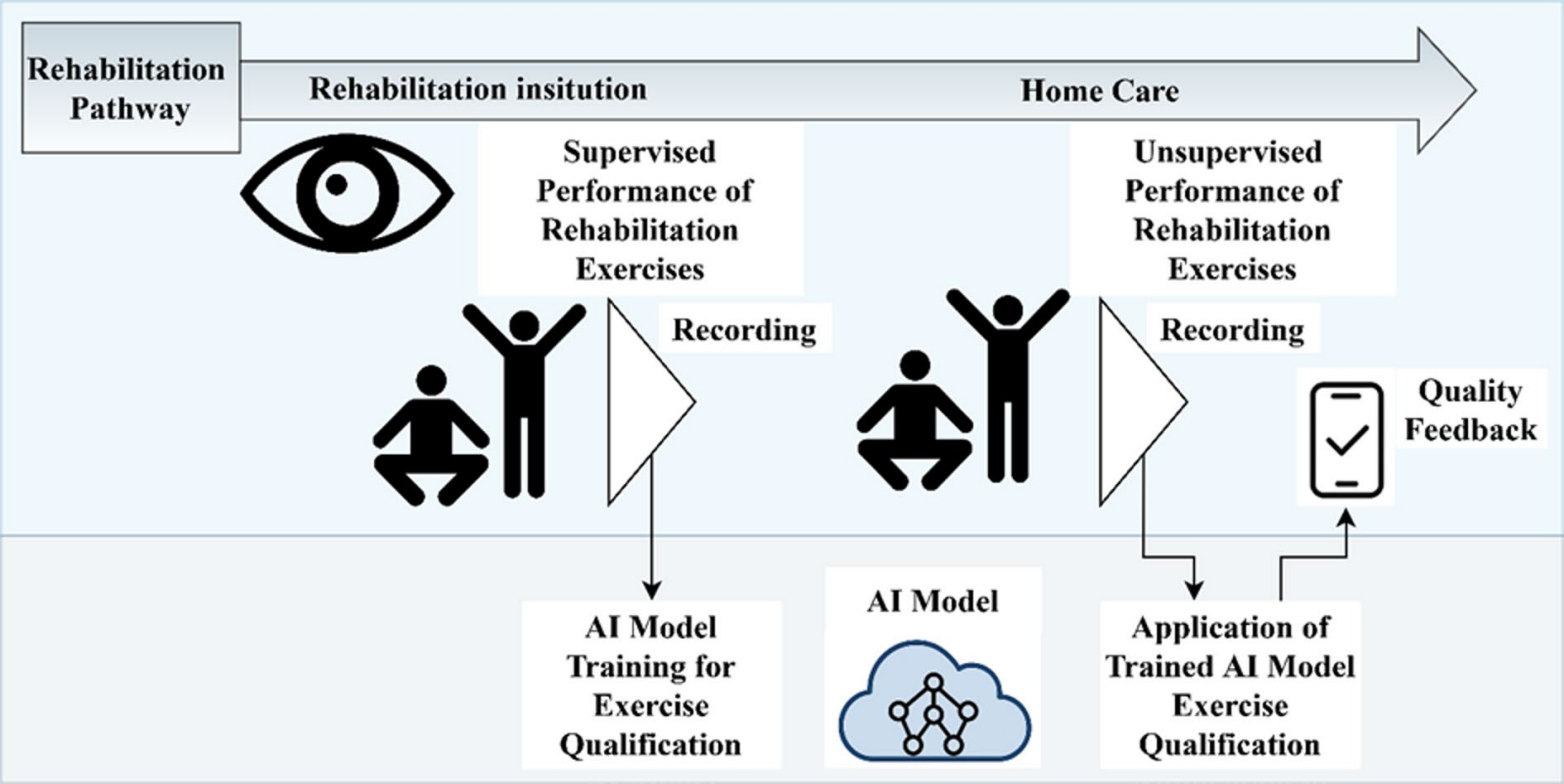
Overview of the proposed system architecture for evaluating home-based rehabilitation exercises using EMG measurements. The first stage involves recording optimal exercise executions and using them to train an ML model. In the second stage, this model is used to analyse newly performed exercises in a home environment and provide feedback on their quality, thereby supporting correct execution and improving rehabilitation adherence.a.

This paper proposes an electromyography (EMG)-based sensor system combined with Machine Learning (ML) to assess exercise execution quality during rehabilitation, with a focus on home-care accessibility. The concept starts with patients performing exercises under supervision generating training data for a ML model. Patients then perform exercises independently at home and the ML model is used to analyse the exercise execution quality and provide personalised feedback ensuring correct execution (Fig. 1).

To evaluate the feasibility of the proposed concept, the following hypotheses are investigated:

### Hypothesis 1

It is hypothesised that EMG recordings of variations of a single exercise contain patterns that can be recognised by a ML model. Once trained, the model should be able to classify unseen EMG recordings as consistent with, or deviating from, the typical neuromuscular activation patterns observed during the correct execution.

This approach enables the supervised classification of exercise execution quality (detecting optimal versus non-optimal executions). While Hypothesis 1 serves as a proof of concept for supervised EMG-based classification, its practical implementation is limited by the need for explicitly labelled data representing both correct and incorrect exercise variants. However, it is difficult to record such a comprehensive dataset in realistic settings, as patients cannot be asked to perform suboptimal rehabilitation exercises.

### Hypothesis 2

It is further hypothesized that a ML algorithm trained exclusively on EMG recordings from optimally executed exercises can detect deviations in unseen recordings.

This is achieved through novelty detection, whereby the model learns an internal representation of the normative activation patterns and identifies variations as potentially erroneous or compensatory. This approach allows for error detection in rehabilitation exercises without requiring annotated examples of incorrect execution, thereby increasing scalability and clinical applicability.

### State of the art

While various technologies are available to quantify rehabilitation exercises, their applicability and precision differ significantly depending on the context:

- Inertial Measurement Units (IMUs) have proven effective for classifying exercise execution^12^, but they have several limitations, including sensor drift, sensitivity to sensor placement and limited feasibility in unsupervised home settings^13,14^.
- Stretch-sensors demonstrate high accuracy in monitoring specific exercise patterns, yet they are restricted in terms of applicability range and often require expert handling and configuration^15^.
- Marker-based motion capture systems offer high-precision but are generally unsuitable for home-based settings due to their high cost and complex setup requirements^16^.

Despite successful evaluations, these technologies are often limited to controlled settings or specific rehabilitation applications. This study therefore investigates the feasibility of an alternative approach based on surface EMG. Here, instead of observing the movement performed, the underlying activation of the muscles is observed. Such an approach could have the potential to reduce the limitations of other modalities and enable broader applicability.

Surface EMG enables non-invasive recording of muscle activity and has been widely used in the fields of biomechanics, rehabilitation and motor control research. EMG signals reflect underlying neuromuscular activation patterns and can capture even subtle deviations in movement execution^17–19^. Our previous research^20^ and findings by Akuzawa et al.^21^ demonstrate that even minor variations in exercises result in measurable changes in EMG signals, emphasising the potential of EMG measurements for assessing exercise quality.

While existing EMG research primarily focuses on classifying distinct movement types, the assessment of exercise execution quality remains underexplored. Shifting the methodological focus towards this aspect is a crucial next step in translating EMG measurements into reliable tools for home exercise monitoring^22,23^.

However, the widespread application of EMG in home-based rehabilitation remains challenging, primarily due to the complex signal processing demands, including noise contamination, movement artefacts and electrode-skin interface issues^24,25^.

The high variability of EMG data requires the use of robust pattern recognition techniques to evaluate the exercise execution quality. Support Vector Machines (SVMs) fulfil this requirement by maximizing the margin of a separating hyperplane in the feature space and enabling both linear and non-linear decision boundaries through kernel functions^17,26–30^. SVMs have consistently outperformed alternative classifiers in EMG applications, such as the classification of knee angles during sit-to-stand tasks^28^, the detection of neuromuscular disorders^29^, and gesture recognition from single-channel EMG data^24^. Consequently, SVMs in combination with EMG measurements have found widespread application in diverse domains, including post stroke rehabilitation^30^, neuromuscular disorder diagnosis^31^ and gesture recognition^26,32,33^. Accordingly, SVMs are employed as the primary classification method in this study.

To benchmark SVM performance, the FreshPRINCE pipeline is employed. This framework automatically extracts 794 features from times series and performs classification using a rotation forest ensemble^34–37^ offering protection against model-specific biases.

A well-known limitation of supervised learning approaches is their requirement for labelled data. Specifically, these methods rely on balanced datasets containing samples of both ‘correct’ and ‘incorrect’ executions. However, it is highly impractical to anticipate and acquire data for all potential biomechanical errors that a patient might make in clinical setting. To address this, two semi-supervised novelty detection algorithms are additionally evaluated, each trained exclusively on correctly executed exercises. One-class SVMs (OC-SVM) define a boundary around the normative data and identify outliers as anomalous^38–41^. Isolation Forest (IF) algorithms detect anomalies by constructing an ensemble of binary decision trees, assigning higher anomaly scores to observations with shorter average path length^28,42^.

## Materials and methods

### Operational definition of exercise execution quality

In this study, ‘exercise execution quality’ is defined as the degree to which a participant’s performance of a rehabilitation exercise matches a biomechanically correct movement. Rather than serving as a clinical assessment of rehabilitation progress (which may include pain levels or mobility), this study evaluates the ability to classify expert-defined exercise variations based on EMG patterns. Consequently, the exercise execution quality is defined by the ability of a ML model to successfully distinguish between normal executions and variations.

### Participants and ethics approval

**Fig. 2:**
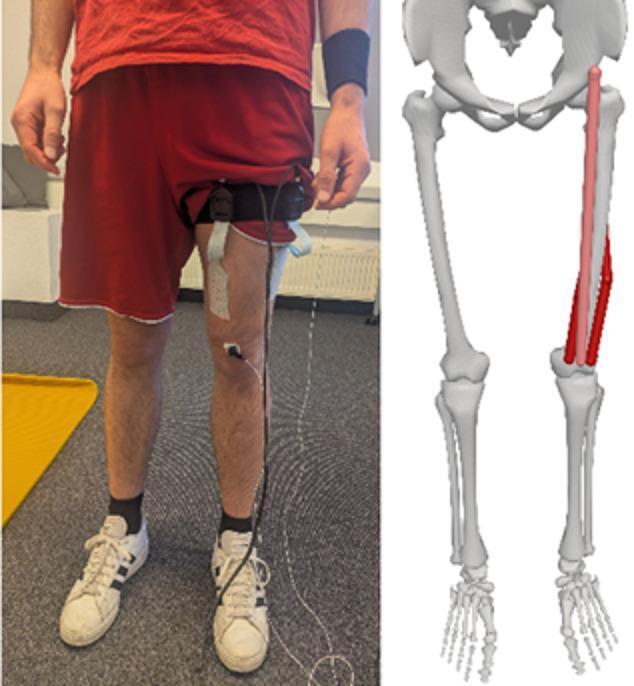
Left: Picture taken during the study showing sensor placement on the vastus lateralis and vastus medialis. Right: Illustration of the positions of the vastus lateralis and vastus medialis, created with OpenSim (https://simtk.org/projects/opensim).

**Fig. 3:**
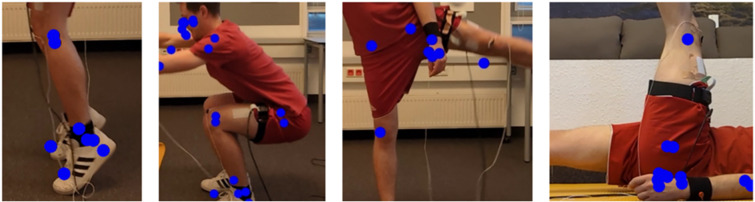
Representative images from the study, depicting one participant performing optimal exercises. (1) Heel and Toe Raises (Rocking); (2) Squat; (3) Hip Abduction; (4) Leg Raises. (Note: The blue dots visible in the frames are artefacts generated by the interface of the recording software and are not relevant to the kinematic analysis or methodology).

**Fig. 4:**
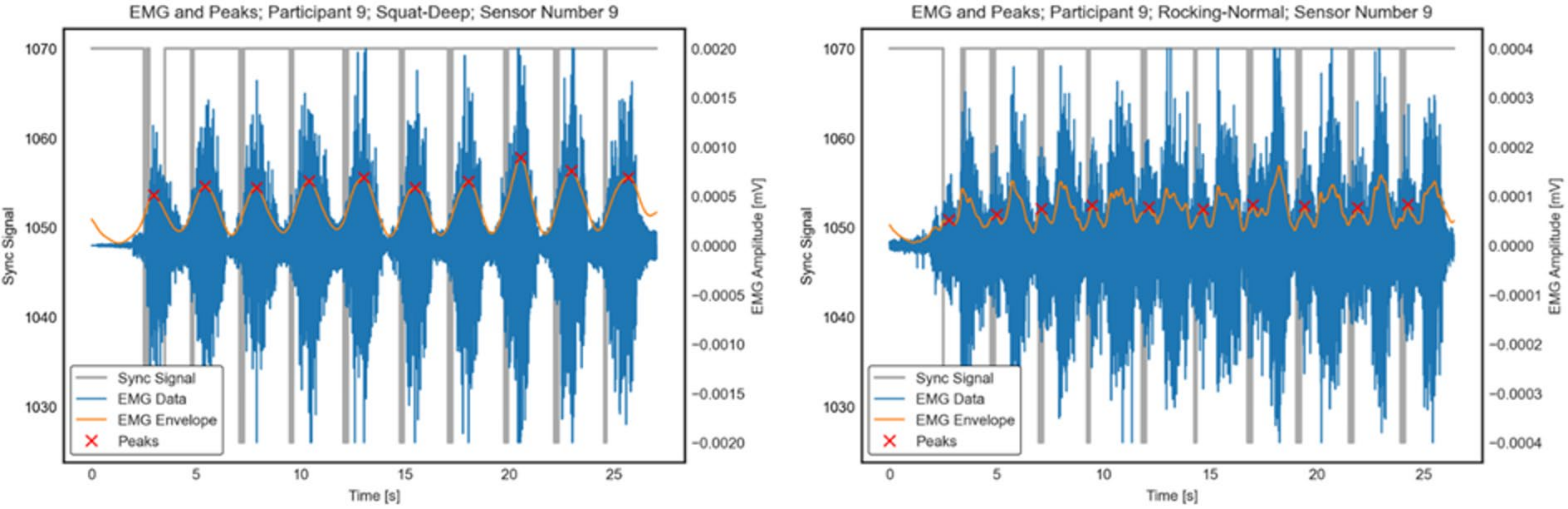
A comparison of peak detection methods for ten repetitions of an exercise. Both plots show the filtered EMG signal in blue and the extracted envelope function in orange. (Left) Peaks identified using an algorithmic peak detection method (red). (Right) Peaks identified using a synchronisation signal based on a button state (grey).

This study was approved by the ethics board of OFFIS e. V. (Ethical vote: 2025G003). All methods were performed in accordance with the Declaration of Helsinki. Participants were recruited via notice boards. All participants were informed that study participation was voluntary and that they could withdraw from the study at any time. After signing informed consent, nineteen healthy individuals were included in the study. To minimise demographic bias, eligibility was restricted to adults aged 18–65 years. Age, gender and weight were not collected to protect participant anonymity; these factors were deemed unnecessary given the exploratory nature of this feasibility study. Exclusion criteria included allergy to tape/silver or implanted electrical devices. Participants also consented for video recording and confirmed their willingness to participate after receiving detailed instructions outlining the exercises. The participant pictured in Figs. 3 and 4 has provided informed consent for an open-access publication of their images.

### Measurement system

Since rehabilitation exercises can place considerable strain on the musculoskeletal system, only a limited number of repetitions can be recorded per participant. This constraint poses a challenge for training ML models, which typically require a certain degree of variability in the training data to ensure generalisability. To overcome this limitation, the present study employed high-density surface EMG (HD-EMG). Recordings were obtained using TMSi sensor pads comprising 64 electrodes, sampled at 4 kHz, with signals acquired in a monopolar (unipolar) configuration (using the knee cap as reference)^43^ (Fig. 2). Although the individual electrodes do not record independent signals, signal variability arises due to signal propagation across muscle and subcutaneous tissue. This spatial richness enables ML models to learn more nuanced patterns of neuromuscular activation, thereby improving generalisation performance even in scenarios with a limited number of repetitions^44^.

The recordings included both optimal executions of the target exercise and deliberately varied modifications. As these classes are highly similar, there may be occasional inconsistencies between the intended class label and the repetition that was actually performed. To mitigate this potential ‘label noise’, video recordings were used to verify and correct class assignments. The recordings were subsequently analysed by two independent researchers using KINOVEA^45^, an open source video analysis tool for frame-by-frame video annotation.

EMG electrodes were placed on the Vastus Lateralis (VL) and Vastus Medialis (VM) muscles (Fig. 2). This choice was motivated by earlier findings, including our own prior work^20^, demonstrating that changes in lower limb movement patterns can be reliably detected by analysing EMG signals from these specific muscles. Additionally, their relevance has been confirmed in related studies^19,46–48^. Sensor placement followed the SENIAM (Surface Electromyography for the Non-Invasive Assessment of Muscles) recommendations for EMG protocols^49^. To enhance signal quality, skin preparation involved shaving the area, cleansing with alcohol, and applying abrasive gel prior to electrode attachment.

### Study design

To evaluate the potential of EMG measurements for assessing exercise execution quality, healthy participants performed four lower-limb exercises (Fig. 3) commonly prescribed in standard postoperative rehabilitation protocols. The correct execution of each exercise served as the ground truth. For each exercise, rehabilitation experts defined three clinically safe variations, based on their professional experience with typical exercise deviations observed in patient populations (Table 1). These variations were designed to simulate representative patterns of suboptimal exercises while ensuring participants safety.

The study was conducted in a controlled laboratory environment. Prior to data collection, all exercises and variations (Table 1) were practised under the supervision of trained researchers to ensure consistent execution. During the experiment, the order of exercises was randomised and each exercise was performed ten times. This minimised potential sequencing effects and mitigated the risk of fatigue accumulation. This precaution is important as Slater and Hart^19^ noted that muscle fatigue can shift EMG frequency content and bias classification results.

**Table 1:** Rehabilitation exercises performed during this study, normal exercises (“ground truth”) and their variations.

Exercise	Ground truth	Variations
Squat	The hips are lowered by bending the knees and then returned to a standing position. The feet should be shoulder wide apart and flat on the floor. Functional for daily activities such as standing up	Less deep (> 100° hip to knee)
		Deeper (< 80° hip to knee)
		Executed on the toes
Heel and Toe raises (Rocking)	Alternates between heel raises (lifting the heels off the floor) and toe raises (lifting the toes). Functional for walking, balance and climbing stairs.	Only heel raises are performed
		Knees are bent
		Pelvis swings forward/backward
Hip Abduction	This involves lifting one leg sideways while standing. The leg remains straight and the body upright without leaning. Functional for balance and walking.	Leg is lifted backwards
		Torso leans opposite to lifted leg
		Leg bent during the raise
Leg Raises	Performed while lying on the back. One leg is lifted straight up without lifting the hips off the floor. Functional for sitting, bending and hip mobility.	Leg is bent during the raise
		Leg not lifted to 90°
		Hip lifts off the floor

### Data preprocessing and feature extracting

The following protocol was used to segment the continuous EMG recordings into discrete epochs, each representing a single exercise execution. The EMG data filtering procedures followed the guidelines published in the *Journal of Electromyography and Kinesiology (JEK)*^50,51^, and employed a Butterworth band-pass filter with cutoff frequencies of 10 Hz (high-pass) and 500 Hz (low-pass).

To further identify repetitions via the EMG signal itself, the absolute value of the raw signal was computed, an envelope was generated, and the result was smoothed using a moving-average filter. Subsequently, peak detection was performed to identify repetition events, as illustrated in Fig. 4 left. Each detected peak was defined as the midpoint of a repetition, and fixed-length segments comprising 4000 data points before and after each peak were extracted for analysis. However, this signal-based approach proved suboptimal when the dominant EMG envelope peak did not align with the actual temporal center of the exercise. For instance, as shown in Fig. 4 right, the prominent peak in the rocking exercise corresponded to the toe-lift (second) phase, a late stage of the action, rather than its initiation. For temporal alignment and accurate segmentation in such cases, a custom synchronisation signal was generated using an Arduino microcontroller, triggered by an external button pressed at the start of each repetition. This signal enabled extraction of the segments even when the EMG peaks were ambiguous.

Resulting in the total number of segments (denoted as $\:{x}_{s,v,r,m}$) per exercise and participant:

$$\:{x}_{s,v,r,m}=2560\:segments\:\left(32\:\left(s\right)x4\left(v\right)x10\left(r\right)x2\left(m\right)\right)$$

With s=sensors, v=variations, r=repetitions, m=muscles (VM/VL).

For the subsequent ML analysis, $\:P=50$ features/predictors were extracted from each time series. The full list of the features is provided in the Appendix. Leading to the following dataset (X) per exercise and participant:

$$\:X=P\times\:{x}_{s,v,r,m};X=50\:predictors\:\times\:2560\:segments\:$$

Furthermore, for Hypothesis 1, the dataset contains C = 4 classes (one standard execution and three variations), while for Hypothesis 2, it contains C = 2 classes (standard execution versus novel exercise execution). This structure aligns with the notation presented by Kuhn and Johnson^28^. One dataset was excluded from analysis due to poor signal quality. Specifically, participant 4 exhibited sporadic signal loss across multiple EMG channels, likely caused by compromised adhesion of the sensor grid.

The execution of each EMG exercise was assigned its corresponding label through a step-by-step process conducted by two independent researchers. First, each video recording of a repetition was visually inspected. For exercises defined by quantitative thresholds (e.g. squats and leg raises), the point of maximum movement displacement was identified and the joint angles were measured at this specific frame using KINOVEA’s angle tool. For exercises evaluated via qualitative thresholds (e.g. hip abduction and heel-and-toe raises), binary visual confirmation of the movement deviation was noted. These measured values and visual observations were then compared against the specific criteria outlined in Table 1 to assign each repetition to its corresponding class. In cases of disagreement between the two researchers, the footage was reviewed together and a mutual agreement reached. Repetitions that could not be reliably assigned to a class were excluded from further processing (in total eleven repetitions were excluded).

### ML for data analysis

To prevent cross-channel data leakage during the model’s evaluation, all 32 EMG channels from a single repetition were treated as a single, coherent unit. This ensures, when the data is split for validation, each repetition is either entirely in the training set or entirely in the test set.

To test Hypothesis 1, two supervised ML pipelines, one based on SVM and the other on the FreshPRINCE approach, were implemented. Both were applied individually to each participant and exercise. The Wilcoxon signed-rank test (implemented in SciPy^52^ was used to compare the results of the SVM and the FreshPRINCE approach. This non-parametric test was chosen in order to account for both the sample size (*n* = 19) and the within-subject cross-validation design. The significance level was set at α = 0.05.

#### SVM supervised classification pipeline

- Data splitting: The data is split into training (nine normal exercises and three times nine variations) and validation (one unknown normal exercise and three unknown variations) subsets, ensuring that exactly one selected repetition is retained as validation data. This process ensures independence between training and validation data, thereby preventing data leakage.
- Feature extraction: Features from the raw EMG signals (see appendix) for each repetition and sensor are extracted and consolidated into structured data frames, each labelled according to its class (zero for normal and one, two and three for variation).
- Model training and hyperparameter tuning: An SVM classifier, including feature standardisation, is trained on the training dataset. Hyperparameter optimisation is performed by grid search with 5-fold cross-validation. Evaluated hyperparameters include kernel type (linear, rbf, poly, sigmoid), regularisation parameter (C) and kernel coefficients (gamma and coef0).
- Evaluation and performance metrics: Model performance is evaluated with every Leave-One-Repetition-Out (LORO) (10 fold, every repetition is tested once), using accuracy (correct predictions over totals predictions), indicating how well the observed and predicted classes match^28^. Additionally, the F1-Score is computed for each exercise variation. The F1-score is a robust measure of a model’s classification ability, with 1 being the best possible score and 0 the worst. It is based on the harmonic mean of Precision and Recall^53^.

$$\:F1\:Score=2\left(\frac{precision\:\times\:\:recall}{precision+recall}\right)$$

Additionally, a limited post-hoc analysis was conducted to quantify the generalizability advantage of HD-EMG. This analysis simulated a single-sensor setup by isolating and evaluating one randomly selected sensor per exercise through an identical SVM pipeline.

#### FreshPRINCE classification pipeline (largely following the structure of the SVM pipeline)

- Feature extraction: Using the tsfresh library and its EfficientFCParameters set, features are automatically extracted. Features are then reduced by selecting only those with significant predictive power based on their p-values obtained from statistical tests. The top 50 most relevant features are used.
- Classification with Rotation Forest: A rotation forest classifier pipeline combined with standardisation is trained and optimised by hyperparameter tuning using grid search with 5-fold internal cross-validation. Hyperparameters optimised include the number of estimators, group size for rotations and PCA solver options.

To test Hypothesis 2, two semi-supervised ML pipelines, one based on OC-SVM and the other on IF, were implemented. Both were applied individually to each participant and exercise. The Wilcoxon signed-rank test was also used to compare the results.

### OC-SVM and IF pipeline

- Data splitting: Normal exercise sets are divided into training data (eight repetitions), validation data (one repetition) and test data (one repetition); in addition, one repetition (randomly selected) of each variation is used as test anomaly data^38^.
- Feature extraction: Features are extracted identically to the pipeline described for Hypothesis 1.
- Model training and hyperparameter tuning: The classifier, including feature standardisation, is trained on the training dataset. OC-SVM hyperparameters (kernel, nu and gamma) and IF hyperparameters (n_estimators, contamination, max_samples, max_features) are optimized over 300 Bayesian trials using Optuna^54^. This maximises the model’s confidence scores on normal validation data.
- Threshold selection: The decision boundary threshold (normal vs. variation) relies on the strictness controlled by the *nu* hyperparameter for OC-SVM, and the *contamination* hyperparameter for IF.
- Evaluation and performance metrics: Model performance is evaluated with every Leave-One-Repetition-Out (LORO) (10 fold, every repetition is tested once). Performance Metrics include balanced accuracy and ROC-AUC (Receiver Operating Characteristic - Area Under Curve)^39,55^. ROC-AUC is a performance metric that evaluates the ability of a classifier to distinguish between *normal* and *anomalous* data^55^. AUC = 1.0 signifying perfect distinction between classes, and AUC = 0.5 corresponding to performance no better than random guessing^28^. Balanced Accuracy accounts equally for correct detection of normal (TNR) and anomalies (TPR).

$$\:Balanced\:Accuracy=\frac{TPR+TNR}{2}$$

$$\:TPR=\frac{TP}{TP+FP}\:;\:TNR=\frac{TN}{TN+FP}$$

With True Positive Rate (TPR), True Negative Rate (TNR), True Positive (TP), True Negative (TN) and False Positive (FP).

## Results

### Hypothesis 1

It is hypothesised that EMG recordings of variations of a single exercise contain patterns that can be recognised by a ML model. Once trained, the model should be able to classify unseen EMG recordings as consistent with, or deviating from, the typical neuromuscular activation patterns observed during the correct execution.

The results of the SVM classification used to evaluate Hypothesis 1 are given in Tables 2 and 3. The Table 2 provides an overview of classification accuracy and standard deviation per participant and exercise (recordings of VL). The Table 3 provides an overview of F1 scores per exercise and averaged over all participants.

**Table 2:** Accuracy and standard deviation per participant from the SVM evaluation for classifying exercise variations (four-class problem: one normal execution and three variations).

*P*.	Squat		Hip-Abduction		Leg-Raises		Toe-heel-raises	
	Accuracy	STD	Accuracy	STD	Accuracy	STD	Accuracy	STD
1	80.6%	18.0%	81.6%	10.0%	74.3%	13.0%	81.3%	10.0%
2	69.0%	14.0%	85.7%	6.0%	89.5%	7.0%	79.2%	17.0%
3	59.0%	23.0%	88.4%	9.0%	98.7%	2.0%	79.1%	16.0%
5	89.6%	10.0%	92.4%	10.0%	88.6%	8.0%	90.6%	9.0%
6	77.9%	18.0%	76.2%	14.0%	75.3%	13.0%	67.7%	9.0%
7	74.2%	14.0%	90.9%	8.0%	89.3%	10.0%	92.7%	7.0%
8	76.3%	21.0%	86.6%	15.0%	89.8%	9.0%	96.0%	6.0%
9	70.8%	13.0%	86.4%	10.0%	89.2%	16.0%	80.8%	9.0%
10	67.0%	12.0%	87.3%	10.0%	82.3%	10.0%	92.3%	9.0%
11	64.3%	6.0%	76.5%	12.0%	91.6%	12.0%	86.5%	11.0%
12	77.0%	9.0%	88.0%	10.0%	76.7%	7.0%	86.5%	11.0%
13	81.5%	13.0%	59.1%	8.0%	89.2%	9.0%	55.6%	10.0%
14	91.3%	10.0%	83.4%	15.0%	90.1%	8.0%	77.3%	13.0%
15	64.8%	13.0%	82.6%	14.0%	88.4%	8.0%	97.6%	4.0%
16	94.9%	7.0%	89.8%	7.0%	86.6%	7.0%	89.1%	9.0%
17	80.6%	18.0%	88.4%	7.0%	92.9%	5.0%	77.6%	12.0%
18	79.1%	8.0%	98.5%	2.0%	96.6%	8.0%	88.0%	10.0%
19	78.0%	15.0%	92.1%	9.0%	88.1%	17.0%	94.1%	7.0%
AV	76.4%		85.2%		87.6%		84.0%	
STD	9.6%		8.5%		6.6%		10.6%	

Recordings of squats and their variations, hip abductions and their variation, leg raises and their variations, and toe–heel raises and their variations were analysed. The average accuracy and standard deviation for each exercise are listed below.

**Table 3:** F1-Score and standard deviation per exercise, averaged over all participants from the SVM evaluation for classifying exercise variations (four-class problem: one normal execution and three variations).

Exercise	F1-Score	Exercise	F1-Score
Hip-Abduction - Back	0.90 ± 0.08	Squat - Deep	0.71 ± 0.15
Hip-Abduction - Bent	0.92 ± 0.08	Squat - Light	0.86 ± 0.11
Hip-Abduction - Normal	0.74 ± 0.17	Squat - Normal	0.66 ± 0.16
Hip-Abduction - Upper Body	0.77 ± 0.12	Squat - Toes	0.70 ± 0.17
Toe-Heel-Raises - Dip	0.87 ± 0.14	Leg-Raises - Bent	0.93 ± 0.07
Toe-Heel-Raises - Normal	0.78 ± 0.13	Leg-Raises - Hip	0.82 ± 0.12
Toe-Heel-Raises - Pelvis	0.76 ± 0.16	Leg-Raises - Light	0.85 ± 0.11
Toe-Heel-Raises - Toes	0.89 ± 0.10	Leg-Raises - Normal	0.86 ± 0.13

Average classification accuracy and standard deviation across all participants (Table 2):

- Squat: 76.4% (± 9.6%).
- Hip-abduction: 85.2% (± 8.5%).
- Leg-raises: 87.6% (± 6.6%).
- Toe-heel-raises: 84.0% (± 10.6%).

The average classification accuracy across all exercises resulted in 83.3% (± 8.8%). The SVM achieved high F1-scores for exercise deviations, whereas normal executions yielded lower F1- scores (Table 3).

To validate the classification accuracy, the FreshPRINCE pipeline was applied, yielding a comparable average accuracy of 83.2% (± 8.6%). A Wilcoxon signed-rank test was performed on the aggregated scores at the subject level. This revealed that the overall difference in model performance was not statistically significant (*p* = 0.580).

When using EMG signals from VM instead of VL, the results were slightly lower: Accuracy over all exercises using SVM = 81.3% (± 8.8%) and using FreshPRINCE = 82.2% (± 8.4%). Consistent with the VL results, the difference between the models was not statistically significant (*p* = 0.130). The performance for the comparison of VM and VL yielded no significant differences for either the SVM (*p* = 0.067) or FreshPRINCE (*p* = 0.325) model.

Binary classification (standard versus variation) was also tested. The SVM achieved a higher average accuracy of 90,1% (± 7.9%) across all exercises and participants.

Finally, the post hoc single-sensor evaluation yielded slightly lower classification accuracies: 80.1% (± 4.9%) for the VL and 79.4% (± 4.6%) for the VM.

### Hypothesis 2

It is hypothesized that a ML algorithm trained exclusively on EMG recordings from optimally executed exercises can detect deviations in unseen recordings,* enabling semi-supervised assessments without the need for labelled variation data.*

An OC-SVM novelty detector, trained exclusively on standard exercise executions, was tested on unseen repetitions of both standard and variated exercises using EMG signals from VL. Table 4 provides an overview of balanced accuracy and ROC-AUC per participant and exercise.

**Table 4:** Accuracy and standard deviation per participant from the OC-SVM evaluation for semi-supervised novelty detection (two-class problem: normal execution vs. variations).

*P*.	Squat		Hip-abduction		Leg-raises		Toe-heel-raises	
	Acc.	ROC- AUC	Acc.	ROC-AUC	Acc.	ROC- AUC	Acc.	ROC- AUC
1	75.2%	0.87	84.4%	0.97	77.7%	0.9	83.0%	0.98
2	73.3%	0.77	81.7%	0.89	80.9%	0.91	84.4%	0.98
3	60.3%	0.72	79.7%	0.88	78.6%	1	72.3%	0.79
5	73.1%	0.9	82.6%	1	83.4%	0.96	85.9%	1
6	84.7%	0.91	75.2%	0.88	76.9%	0.88	58.9%	0.61
7	71.7%	0.81	79.7%	0.9	77.2%	0.8	75.0%	0.89
8	65.1%	0.77	76.0%	0.94	79.1%	0.95	78.8%	0.98
9	58.2%	0.65	79.1%	1	77.8%	0.92	73.8%	0.77
10	83.3%	0.96	81.6%	0.95	78.4%	0.88	74.8%	0.91
11	68.0%	0.75	78.9%	0.88	81.3%	0.97	79.7%	0.94
12	73.0%	0.87	79.5%	0.97	78.1%	0.88	69.8%	0.68
13	74.5%	0.84	67.5%	0.75	79.7%	0.98	71.1%	0.74
14	79.8%	0.99	80.3%	0.96	69.8%	0.82	80.9%	1
15	74.0%	0.83	72.0%	0.8	68.0%	0.84	78.7%	0.84
16	75.6%	0.82	83.6%	1	82.3%	0.9	88.4%	1
17	61.7%	0.64	75.8%	0.88	81.7%	0.91	73.4%	0.83
18	66.3%	0.75	88.8%	1	81.1%	0.96	80.6%	1
19	61.7%	0.72	77.0%	0.98	84.7%	1	85.2%	1
AV	71.1%	0.81	79.1%	0.92	78.7%	0.91	77.5%	0.89
STD	7.7%	0.10	4.8%	0.07	4.2%	0.06	7.2%	0.12

Recordings of squats and their variations, hip abductions and their variation, leg raises and their variations, and toe–heel raises and their variations were analysed. The average accuracy and standard deviation for each exercise are listed below.

The average balanced accuracy and ROC-AUC for each exercise and across all participants was:

- Squat: 71.1% (± 7.9%) with a corresponding ROC-AUC of 0.81 (± 0.10).
- Hip-abduction: 79.1% (± 4.8%) with a corresponding ROC-AUC of 0.92 (± 0.07).
- Leg-raises: 78.7% (± 4.2%) with a corresponding ROC-AUC of 0.91 (± 0.01).
- VL-Toe-heel-raises: 77.5% (± 7.2%) with a corresponding ROC-AUC of 0.89 (± 0.12).

The average accuracy across all exercises resulted in 76.6% (± 5.9%), when detecting unseen exercise variations as either ‘optimal’ or ‘novelty’ based on VL measurements (Table 4).

The average balanced accuracy using recording from the VM resulted in slightly lower accuracy of 74.8% (± 2.1%). A Wilcoxon signed-rank test confirmed that the improvement observed within subjects when using VL data was highly statistically significant (*p* < 0.001).

An IF novelty detector, trained exclusively on standard exercise executions, was tested on unseen repetitions of both standard and variated exercises using EMG signals from VL. The average balanced accuracy and ROC-AUC for each exercise and across all participants was:

- Squat: 73.2% (± 10.7%) with a corresponding ROC-AUC of 0.83 (± 0.10).
- Hip-abduction: 80.5% (± 10.4%) with a corresponding ROC-AUC of 0.92 (± 0.06).
- Leg-raises: 78.6% (± 8.3%) with a corresponding ROC-AUC of 0.90 (± 0.07).
- Toe-heel-raises: 83.3% (± 12.8%) with a corresponding ROC-AUC of 0.91 (± 0.11).

The overall average balanced accuracy (VL) resulted in 78.9% (± 10.6%) when detecting unseen exercise variations as either ‘optimal’ or ‘novelty’ based on VL measurements.

Using signals from the VM, the overall average balanced accuracy across all exercises was slightly lower at 75.9% (± 5.7%). Wilcoxon signed-rank test confirmed that this performance advantage was statistically significant (*p* = 0.010).

The performance for the comparison of OC-SVM and IF yielded significant differences for the VL (*p* = 0.034) and no significant differences for the VM (*p* = 0.196).

## Discussion

Performing ten repetitions per exercise is consistent with common study designs in related literature. For example, Dhindsa et al.^46^ included ten repetitions per set to evaluate the performance of different ML approaches in classifying stand-up exercises. Likewise, the sample size in the present study aligns with previous EMG-based research: Dhindsa et al.^46^ included twelve participants to compare the effectiveness of different ML approaches on classification of stand-up exercises; Ahlawat et al.^33^ included 15 healthy participants when classifying hand movements.

### Hypothesis 1

It is hypothesised that EMG recordings of variations of a single exercise contain patterns that can be recognised by a ML model. Once trained, the model should be able to classify unseen EMG recordings as consistent with, or deviating from, the typical neuromuscular activation patterns observed during the correct execution.

Using a SVM resulted in an average accuracy of 83.3% (± 8.8%), significantly exceeding 25% chance level. This confirms the algorithm’s ability to classify optimal exercises executions and their variation, indicating its potential to predict rehabilitation exercise execution quality.

Other studies report comparable results. Tryon et al.^56^ reported an accuracy of 74.7% when classifying EMG signals into three categories related to elbow flexion while holding different weights. Guo et al.^25^ achieved an accuracy of 62.97% when using a SVM to classify five different gait patterns (normal, in-toeing, out-toeing, supination, and overpronation). They increased their accuracy by working with Discrete Wavelet Transform coefficients in combination with Bidirectional Long Short-Term Memory networks. Such hybrid models might offer promising extensions for future work. That said, direct comparisons remain difficult, as the classification of exercise quality via EMG is a relatively novel approach.

One finding of this study is that the performance of the SVM baseline is comparable to that of the FreshPRINCE pipeline. The absence of statistically significant differences between the two models for both VL (*p* = 0.580) and VM (*p* = 0.130) inputs shows that a traditional classification approach can match the performance of a modern algorithm. Furthermore, the absence of significant differences in performance between VM and VL sensor placements (*p* = 0.067 and *p* = 0.325, respectively) highlights the robustness of the system. This indicates that high accuracy can be maintained even with variations in muscle targeting or sensor positioning.

The results show high standard deviations (Table 2), indicating differences in performance. For some participants the models experienced significant losses in performance in certain exercises (e.g. Participant 3 achieved 59.0% in squats, but 98.7% in leg raises). A major factor in these subject-specific declines is the natural ambiguity in the execution of movement variations. Since there is no absolute ‘fundamental truth’ for an incorrect exercise, participants perform these variations with varying degrees of severity. Some executions are exaggerated, while others are very similar to the normal movement, which naturally leads to difficulties in classification. Furthermore, variability between subjects is exacerbated by the fundamental nature of EMG measurements, whereby individual differences in tissue, muscle activation strategies and discrepancies in sensor placement relative to the muscle belly influence the final signal and model performance^57^. The high standard deviation in classification results are consistent with observations reported in other studies^56,58^.

The resulting F1-scores indicate that the model performs robustly when classifying distinct biomechanical deviations, but struggles with variations that share highly similar muscle activation patterns with the normal execution (Table 3). This suggests that the algorithm more easily detects pronounced deviations in exercise execution quality than subtle variations.

In this study, HD-EMG grids were used for their potential to improve generalizability. To verify this advantage, a post hoc analysis was conducted in which only one randomly selected sensor per exercise was evaluated using the SVM pipeline. As expected, the classification accuracy was slightly lower than that of the full sensor array: 80.1% (± 4.9%) for the VL and 79.4% (± 4.6%) for the VM (four-class problem, averaged across all exercises and participants). These results confirm that HD-EMG grids provide superior generalizability, but also indicate that single-sensor systems may suffice for basic exercise execution quality monitoring.

### Hypothesis 2

It is hypothesized that a ML algorithm trained exclusively on EMG recordings from optimally executed exercises can detect deviations in unseen recordings, enabling semi-supervised assessments without the need for labelled variation data.

Both novelty detectors performed best using VL data. The IF achieved an average balanced accuracy of 78.9% (± 10.6%), while the OC-SVM achieved 76.6% (± 5.9%). Although the IF demonstrated a statistically significant improvement over the OC-SVM when utilising the VL signals (*p* = 0.034), the absolute increase in accuracy was modest (approximately 2.3%). From a practical perspective, the IF algorithm with VL sensor placement achieves the best performance. However, the real-world differences between the two approaches are marginal. Furthermore, utilising VM signals resulted in statistically significantly lower performance than VL signals, but the absolute difference remained marginal. This demonstrates the models’ robust generalisability across different muscle inputs.

In strict semi-supervised contexts, the ideal decision threshold is unknown. As the models were trained exclusively on ‘standard’ movement data, using ‘variation’ data to optimise this threshold empirically would have violated the semi-supervised premise. However, the results indicate that the heuristically chosen thresholds were reasonably accurate for most instances: in 80% of cases, the difference between the ROC-AUC and balanced accuracy was less than 20%, and in 55% of cases, it was less than 10%. This shows the robustness of the model. Conversely, the ROC-AUC exceeded balanced accuracy by more than 20% in only 20% of cases, and by more than 10% in 45% of cases. These cases demonstrate instances where the threshold was chosen suboptimally and show that absolute performance could be improved by an ideal boundary. In practice, to address this limitation, the overall performance of the models could be improved simply by expanding the training data. This would enable the models to identify a more precise optimal exercise execution boundary.

These findings highlight the challenges and potential of using novelty detection models for assessing rehabilitation exercise execution quality.

### Limitations

As an initial proof-of-concept study, this work evaluates the technical feasibility of using EMG to assess exercise execution quality. Consequently, as the study was not designed as a clinical trial, a formal prospective statistical power analysis was not conducted to determine the required sample size. A limitation of this feasibility study is its reliance on a cohort of healthy participants. Individuals undergoing active clinical rehabilitation may exhibit different baseline EMG patterns due to factors such as pain, muscle atrophy or compensatory motor control strategies. This necessitates the future validation of these models on specific patient populations. Another limitation is the size of the dataset, particularly the small number of repetitions of normal exercises, which restricts the performance of novelty detection models.

Furthermore, only three predefined exercise variants were included in this study protocol. This controlled approach was necessary to evaluate the classification capabilities of the algorithms, as defined variations made it possible to verify whether the ML models could also classify the type of variation. However, we acknowledge that these predefined variants do not adequately represent the heterogeneous movements that occur in clinical reality. Consequently, the ecological validity of the current data set is limited. Future studies must evaluate these models using unstructured, real-world variations in order to assess their clinical robustness.

Additionally, data were collected in a controlled laboratory environment using a high-density EMG system. This system offers high performance and multiple measurements of the same movement, making it suitable for an initial feasibility study. However, the setup is impractical for home use, which currently limits the generalisability of our findings. Nevertheless, our analysis of a single-sensor configuration demonstrated that alternative, more accessible systems could realistically be used at home.

Finally in a real-world scenario, EMG sensors would repeatedly be taken on and off, which would introduce signal variation due to changes in sensor placement, skin impedance, and the physiological state of the muscle. As only continuous sessions were recorded in this study, cross-session performance was not assessed, which is a major limitation. Evaluating and mitigating this cross-session variability will be a crucial next step towards EMG sensor usage in monitoring exercise execution quality.

## Conclusion

This study demonstrates the feasibility of using ML methods to assess exercise (squats, leg-raises, rocking and hip-abduction) execution quality during rehabilitation based on EMG data. In total, 3040 exercise executions were analysed. Specifically, supervised ML algorithms were applied to classify EMG recordings from the VL and VM muscles into optimal execution and three variations, achieving an average accuracy of 83.3%, thus providing information on exercise execution quality. When simplifying the classification to a binary problem (optimal versus non-optimal performance), the average accuracy increased to over 90%, underlining the clinical potential of this approach. The achieved accuracy represents a promising first step toward implementing the proposed concept (Fig. 1).

In addition, semi-supervised algorithms such as OC-SVM and IF, trained using only optimally executed exercises, were able to detect previously unseen deviations with a balanced accuracy of 78.9%, thereby eliminating the need to collect training data of incorrect movements.

Overall, this work highlights the potential of combining surface EMG with ML-based signal analysis to enable the automated and objective evaluation of exercise execution quality during rehabilitation. While such systems could eventually support patients through real-time feedback and improve exercise adherence, it must be emphasized that this was a feasibility study, conducted in a controlled laboratory setting. Further research and extensive out-of-the-lab validation are required to translate these preliminary findings into scalable, robust systems suitable for real-world clinical or home-based rehabilitation.

## Data Availability

The data supporting the findings of this study consist of video recordings of the participants. Due to privacy concerns, these data are not publicly available. However, they are available upon request from the corresponding author.

## Appendix

This paper does not evaluate the quality of the extracted features; they are merely used as input for ML models. Therefore, a detailed explanation is not provided, but relevant sources offering further information are included.Time-Domain Features: Average Amplitude Value^59^; Mean Absolute Value^18,59,60^ Maximum amplitude^60,61^; Minimum Amplitude^60,61^; Root Mean Square^46,60–62^; Variance^61^; Standard Deviation^60^;Integrated EMG^46,61^; Simple Square Integral^59^; Experimental Variogram (lag variance)^59^; Derivative Standard Deviation (smoothness)^63^; Sign change count^62^; Zero-crossing count^46,62^; Log-mean signal amplitude^64^; Myopulse percentage (> threshold)^59^; Willison amplitude (> threshold)^46,62^; Avg. autoregressive coefficient^59^Frequency- Domain Features: Mean Frequency^46,65^; Peak Frequency^46^; Mean Power^46^; Total Power^59^; Median Frequency^46,65^; Mean normalized power^46^, Variance of central frequency^66^; Frequency Ratio^59^; Power spectral ratio^59^; Power Spectral Density^59^; Frequency-domain entropy^67^ Cepstral Coefficients (1-5)^59^Time-Frequency Features: Short Time Fourier Transform^68^; Continuous wavelet coefficients^59^; Wavelet packet energy^59^Spectral & Window Features^59^: Hamming/Bartlett windowed mean amplitude; Blackman-windowed mean amplitude; Hanning-windowed mean amplitude; Gaussian-windowed mean amplitude; Kaiser-windowed mean amplitudeHjorth Parameters^63^; Hjorth Activity; Hjorth Mobility; Hjorth ComplexityNonlinear & Statistical Features: Teager–Kaiser energy (inst. power)^69^; Asymmetry of signal distribution (Skewness)^70^; Peakedness of signal distribution (Kurtosis)^70^; Fractal Dimension (Higuchi method)^71^.
